# OP Pesticides in Children’s Bodies: The Effects of a Conventional versus Organic Diet

**Published:** 2006-02

**Authors:** Julia R. Barrett

Conventional agriculture includes the use of pesticides to control insects in vegetable, fruit, wheat, and other crops, so it’s no surprise that foods derived from these crops can therefore contain pesticide residues. What’s in question, though, is what these exposures amount to in terms of body burden. Risk-defining data are lacking, and scant data exist on diet-derived pesticides levels in children’s bodies. Now researchers from Seattle and Atlanta characterize the relationship between eating a diet of conventionally grown food products and the amount of organophosphorus (OP) pesticide residues that make it into children’s bodies **[*EHP* 114:260–263]**.

According to a 1993 National Research Council report titled *Pesticides in the Diets of Infants and Children*, diet delivers the bulk of children’s exposure to pesticides. This exposure poses a greater health risk to children as compared to adults, because not only do children consume more food on a per-weight basis than adults and consequently have higher exposure, they also may be more vulnerable to the effects of toxicants because they are still developing.

The researchers employed a longitudinal design in which 23 children aged 3 to 11 years accustomed to eating a conventional diet switched to organic foods and back again during a 15-day study period. For the first three days, the children consumed their regular conventional diets. During the next five days, they substituted organic equivalents of their usual plant-derived food items (including fresh produce, juice, processed fruits and vegetables, and grain-based products). For the last seven days, they resumed their conventional diets. Each day, for the entire 15-day period, parents collected a urine sample in the morning when the children woke and again at bedtime.

The urine samples were analyzed for metabolites of several OP pesticides. The most commonly detected metabolites were MDA (a metabolite of malathion) and TCPY (a metabolite of chlorpyrifos). During both conventional phases, 60% of samples contained MDA, and 78% of samples contained TCPY. When children switched to organic foods, the percentage of samples containing MDA dropped to 22% and the proportion with TCPY fell to 50%.

Average concentrations of MDA and TCPY also were significantly lower during the organic phase compared to the conventional phases. During the two conventional phases, mean urinary MDA concentrations were 2.9 and 4.4 micrograms per liter (μg/L) compared with 0.3 μg/L in the organic phase. The mean TCPY level decreased from 7.2 to 1.7 μg/L between the first and second phases, and rose to 5.8 μg/L when the children resumed their conventional diets.

Metabolite levels varied widely among the samples, however. Recent research suggests that fractions of MDA and TCPY form as the parent compounds degrade in foods and the environment. Therefore, some proportion of the children’s exposure may have been to the metabolites themselves in the foods.

The current study provides insight into how residual OP pesticides in food correspond with the absorbed dose, and the researchers conclude that a diet of organic foods protects children from exposure. They caution against applying results to the general population, however. Given that people from different backgrounds and living in different areas may have different and more significant OP exposures, it would be a mistake to assume that switching to an organic diet would eliminate all exposure to these pesticides. The study does support the National Research Council’s conclusion that dietary intake is a major source of OP pesticide exposure, but some children may receive even more exposure from the use of pesticides in the home, and further research is needed.

## Figures and Tables

**Figure:**
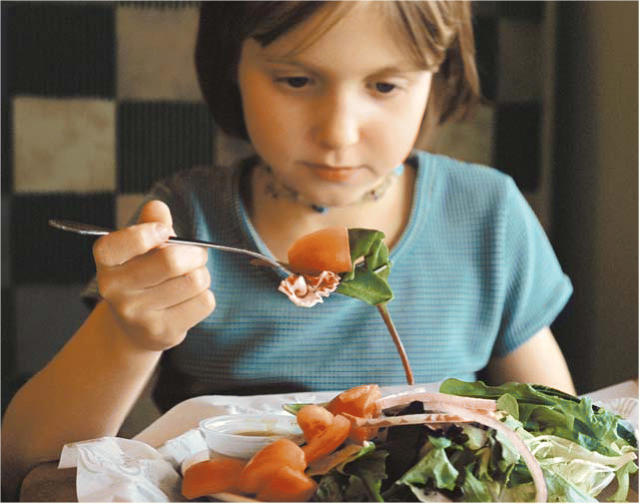
No beef here. A study of organophosphate metabolites in children eating an alternating conventional/organic/conventional diet shows that eating organic plant-derived foods really can reduce pesticide exposure.

